# Longitudinal alterations in health-related quality of life and its impact on the clinical course of patients with advanced hepatocellular carcinoma receiving sorafenib treatment

**DOI:** 10.1186/s12885-016-2908-7

**Published:** 2016-11-11

**Authors:** Masako Shomura, Tatehiro Kagawa, Haruka Okabe, Koichi Shiraishi, Shunji Hirose, Yoshitaka Arase, Kota Tsuruya, Sachiko Takahira, Tetsuya Mine

**Affiliations:** 1 Department of Nursing, Tokai University School of Health Sciences, Isehara, Kanagawa Japan; 2 Division of Gastroenterology, Department of Internal Medicine, Tokai University School of Medicine, Isehara, Kanagawa Japan; 3 University of Nagasaki Department of Nursing, Nagasaki, Nagasaki Japan; 4 143 Shimokasuya, Isehara-city, Kanagawa 259-1193 Japan

**Keywords:** Advanced hepatocellular carcinoma, Sorafenib, Health-related quality of life, Clinical course, Prognostic marker

## Abstract

**Background:**

This study aimed to identify the health-related quality of life (HRQOL) domains associated with prognosis by assessing longitudinal alterations in HRQOL in patients with advanced hepatocellular carcinoma receiving sorafenib.

**Methods:**

We prospectively assessed HRQOL by administering the SF-36 questionnaire 3-monthly to consecutive patients with advanced hepatocellular carcinoma receiving sorafenib. We evaluated the impact of HRQOL on their overall survival and duration of treatment with sorafenib using Cox's proportional hazards model.

**Results:**

There were 54 participants: 42 (78 %) were male, the median age was 71 years, 24 (44 %) had hepatitis C virus infection, 33 (61 %) had Child-Pugh scores of 5, and 30 (56 %) had TNM stage IV hepatocellular carcinoma. The median overall survival and treatment duration were 9 and 5 months, respectively, and 40 patients (74 %) died. Thirteen patients receiving sorafenib over a 1-year period maintained all domain scores >40, without a significant decline during the treatment period. In contrast, physical functioning, physical role, and vitality scores declined continuously and significantly in the year before death (in the 40 patients who died). Previous curative treatment and physical functioning scores ≥40 at baseline were significantly associated with longer overall survival by multivariate analysis. Social functioning scores ≥40, absence of vascular invasion, and lower DCP value were significant predictors of longer treatment duration.

**Conclusions:**

HRQOL was not significantly impaired in those patients who were able to complete a 1-year course of sorafenib treatment. Baseline physical functioning scores ≥40 and social functioning scores ≥40 were significantly associated with longer overall survival and longer treatment duration, respectively. Thus, HRQOL could be a valuable marker to predict the clinical course of patients with advanced hepatocellular carcinoma receiving sorafenib.

**Electronic supplementary material:**

The online version of this article (doi:10.1186/s12885-016-2908-7) contains supplementary material, which is available to authorized users.

## Background

Liver cancer is the third leading cause of cancer-related deaths worldwide [1]. The prognosis of patients with hepatocellular carcinoma (HCC) is poor because of the high recurrence rate and/or the presence of underlying chronic liver disease(s). Sorafenib, a small molecular inhibitor of several tyrosine protein kinases—vascular endothelial growth factor receptor, platelet-derived growth factor receptor, and Raf kinases—extends the median overall survival by nearly 3 months compared with placebo in patients with advanced HCC [2, 3]. However, adverse effects (such as hand-foot skin reactions, diarrhea, or weight loss) and the deterioration in liver function associated with its use, and progressive disease, limit the efficacy of sorafenib.

Quality of life is a multi-dimensional concept that includes subjective evaluation of both physical and mental aspects of life. More specifically, the term “health-related quality of life” (HRQOL) refers to a multidimensional concept that encompasses patients’ perceptions of both negative and positive aspects of at least four dimensions: physical functioning, emotional well-being, social well-being, and spiritual well-being; and disease and treatment-related symptoms. HRQOL assessment is becoming an important component of health surveillance and an indicator of service needs and intervention outcomes. Furthermore, HRQOL could be used as a prognostic marker for patients with various types of cancer [4]. In particular, the baseline physical functioning domain has been associated with survival in patients with non-small cell lung cancer [5] and tumor-node-metastasis (TNM) stages III and IV colorectal cancer [6]. A recent study revealed that physical well-being, evaluated using the Functional Assessment of Cancer Therapy-Hepatobiliary, could be used as a prognostic marker in patients with various stages of HCC and cholangiocarcinoma [7]. However, few studies have explored the association of HRQOL and prognosis in patients with HCC. Targeted molecular therapy, now widely used for many types of cancers, is often accompanied by unique adverse effects, such as hand-foot skin reaction [8]. In terms of adverse effects, longitudinal follow-up for HRQOL is likely to facilitate clinical decision-making by correctly evaluating the patient’s condition. Several studies have reported on changes in HRQOL in patients receiving targeted molecular therapy [9–12], but with controversial results. Sorafenib treatment was associated with a significant decrease in quality of life because of adverse effects in patients with HCC [12, 13] and advanced renal cell carcinoma [12]. In contrast, Miyake et al. could not identify a significant influence of sorafenib on HRQOL in patients with metastatic renal cell carcinoma [9]. Therefore, further evidence on the impact of sorafenib therapy on quality of life and clinical course is required.

In this study, we aimed to clarify longitudinal alterations in HRQOL in patients with advanced HCC [3] receiving sorafenib and to identify the HRQOL domains associated with prognosis.

## Methods

### Patients

We enrolled consecutive patients with advanced HCC who received sorafenib therapy from August 4, 2010 to April 7, 2015 at Tokai University Hospital. Eligibility criteria were as follows: (1) non-resectable advanced HCC; (2) resistance to, or no indication for, transcatheter arterial chemoembolization; (3) Child-Pugh class A or B [14]; (4) TNM criteria of Liver Cancer Study Group of Japan stage III or IV [15]; and (5) Eastern Cooperative Oncology Group Performance Status (ECOG PS) 0 or 1 [16]. Most patients received 800 mg of sorafenib as an initial dose, but lower doses (including 200, 400, and 600 mg) were administered to certain patients, particularly those aged > 70 years or with Child-Pugh class B liver function. Nurses provided educational instructions when initiating sorafenib treatment and gave medical advice by telephone.

### Health-related quality of life assessment

We prospectively assessed HRQOL using a Japanese version of the short form health survey (SF-36) v2™ [17]. We chose SF-36 because its Japanese national standard score was available. Patients completed this questionnaire every 3 months by self-report during their clinic visits. The questionnaire assessed eight domains for health status, with 36 questions covering both mental and physical aspects of health. These aspects included physical functioning; role limitations because of physical problems—referred to as role physical (RP); bodily pain; general health; vitality; social functioning; role limitations because of emotional problems—referred to as role emotional (RE); and mental health. Each domain was scored on a scale of 0–100, with lower scores indicating poorer health status. A score of 50 points, considered the Japanese national standard, was used for comparison with study samples [17, 18]. In this study, we chose a score of 40 points—80 % of the Japanese national standard—as the cut off value.

### Clinical evaluation

Tumor measurements were performed using dynamic computed tomography (CT) or magnetic resonance imaging (MRI) before and every 3 months after initiation of sorafenib treatment. Assessment of disease control and progression was based on the modified Response Evaluation Criteria in Solid Tumors [19]. The disease control rate was defined as the percentage of patients with complete response, partial response, and stable disease 3 months after initiation of sorafenib. Adverse events were evaluated monthly using National Cancer Institute Common Toxicity Criteria (version 4.0) [20]. Patients were followed up until May 25, 2015 or death. The discontinuation of sorafenib was defined as the outcome for treatment duration in this study.

### Statistical analysis

To analyze the changes in HRQOL scores, we used the Friedman test, Bonferroni correction, and Wilcoxon two-sample test for patients who continued sorafenib treatment over a 1-year period, and the Kruskal-Wallis or Mann-Whitney *U* test for the 40 deceased patients. We analyzed the relationship between HRQOL, baseline characteristics, overall survival, and treatment duration using multiple logistic regression and Cox's proportional hazards regression model. Multivariate analysis was performed using the forward stepwise procedure (likelihood ratio). We also analyzed treatment discontinuation incidence using a competing risks approach (Gray’s method). Medians and interquartile ranges (IQR) or means and standard deviations were used to describe non-parametric and parametric data, respectively. Categorical variables were represented in terms of proportions and frequency tables. *P* values < 0.05 were considered to indicate statistical significance. Statistical analysis was performed using IBM® SPSS® statistical software, version 23 for Windows (2015, Somers, NY).

## Results

### Baseline patient characteristics

Of the 54 patients participating in the study, 42 (78 %) were male (Table 1). The median age was 71 years (range, 57–84 years). Nearly half of the patients (44 %) had hepatitis C virus (HCV) infection. Most patients (61 %) had a Child-Pugh score of 5 (median score: 5.0 IQR: 5.0–6.0), and most (57 %) had TNM stage IV HCC. The majority of patients (68 %) had received curative therapy, such as surgical resection and radiofrequency ablation, before enrolment to the study.

**Table 1:** Baseline demographic and clinical characteristics

Variable		*n* (%)
Age, years	<70 ≥70	24 (44) 30 (56)
Sex	Male Female	42 (78) 12 (22)
Etiology	HCV HBV Alcohol-related Unknown	24 (44) 11 (20) 7 (13) 12 (23)
Child-Pugh score, points	5 ≥6	33 (61) 21 (39)
TNM staging	III IV	23 (43) 31 (57)
Vascular invasion	- +	40 (74) 14 (26)
Maximum tumor size, mm	<50 ≥50	33 (61) 21 (39)
Previous therapy	Curative Other None	35 (65) 14 (26) 5 (9)
Serum alpha fetoprotein, ng/mL	<100 ≥100	25 (54) 29 (46)
DCP, mAU/mL ^a^	<1000 ≥1000	27 (52) 25 (48)
Initial dose of sorafenib, mg	200 400 600 800	10 (19) 14 (26) 6 (11) 24 (44)

*DCP* des-gamma-carboxy prothrombin, *HCV* hepatitis C virus, *HBV* hepatitis B virus

^a^DCP values were not available for two cases

We analyzed the association between each HRQOL domain score and patient characteristics at baseline. Female sex was associated with lower physical functioning domain scores than male sex (OR 0.167, 95 % CI 0.039–0.715, *p* = 0.016). Patients aged < 70 years had significantly lower scores in the domain of general health (OR 0.280, 95 % CI 0.080–0.984, *p* = 0.047). Role physical domain scores was significantly higher the patients with previous curative treatment (34.8 ± 11.5 [mean ± SD] points) than without (31.1 ± 17.3 points). Other domain scores were not significantly different in terms of baseline patient characteristics.

### Treatment efficacy and adverse events

Disease control was obtained in 27 patients (50 %): one (2 %) had complete response, five (9 %) had partial response, and 21 (39 %) had stable disease. In total, 40 patients (74 %) died. Almost all patients (98 %) experienced adverse events (Additional file 1: Figure S1). Common adverse events included anorexia (76 %), skin toxicity (72 %), fatigue (63 %), diarrhea (56 %), and weight loss (52 %). There were no grade 4 or 5 adverse effects. Grade 3 adverse effects occurred in 27 patients (50 %). Of these, skin toxicity was most common (33 %), followed by anorexia (15 %) and fatigue (9 %). Sorafenib was discontinued or the dose was reduced in 11 (20 %) and 16 patients (30 %), respectively, because of adverse events. At the end of observation period, sorafenib was withdrew in 42 patients. The reasons for withdrawal were as follows: disease progression (34 patients); adverse events of skin toxicity and diarrhea (two patients); complications such as stroke, pneumonia, and intracranial hemorrhage (three patients); and at the patient’s request (three patients).

### Changes in HRQOL domain scores

Overall, 13 patients (24 %) were able to take sorafenib over the course of at least 1 year. These patients maintained HRQOL domain scores >40 without any significant decline (Additional file 1: Figure S2 and Table S2). Domain scores were not significantly different between patients who experienced grade 3 adverse effects and those who did not (Additional file 1: Figure S3).

**Fig. 1:**
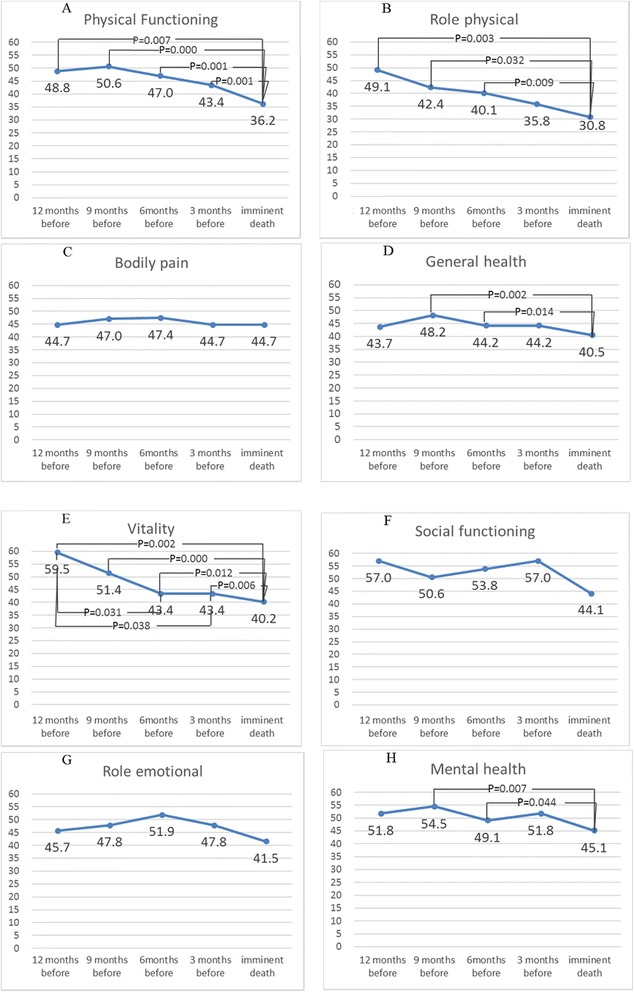
Time-point comparison of changes in HRQOL domain scores in the 12 months prior to death. 12 months before, 12 months before death (*n* = 8); 9 months before, 9 months before death (*n* = 4); 6 months before, 6 months before death (*n* = 22); 3 months before, 3 months before death (*n* = 31); imminent death, less than 3 months before death (*n* = 40). * All values shown are median scores. ** Upper lines show statistically significant differences using Mann-Whitney *U* test (*P* < 0.05). HRQOL, health-related quality of life

Next, we analyzed the changes in HRQOL domain scores over the 12 months preceding death in the 40 patients who died (Fig. 1 and Additional file 1: Table S1).

The psychosocial and pain domains—including bodily pain, general health, social functioning, and mental health—did not demonstrate significant changes, but the scores remained >40 points until imminent death. In contrast, physical functioning, role limitations because of physical problems, and vitality domain scores declined continuously and significantly towards death.

### The association of HRQOL domain scores and patient characteristics with overall survival and treatment duration

**Fig. 2:**
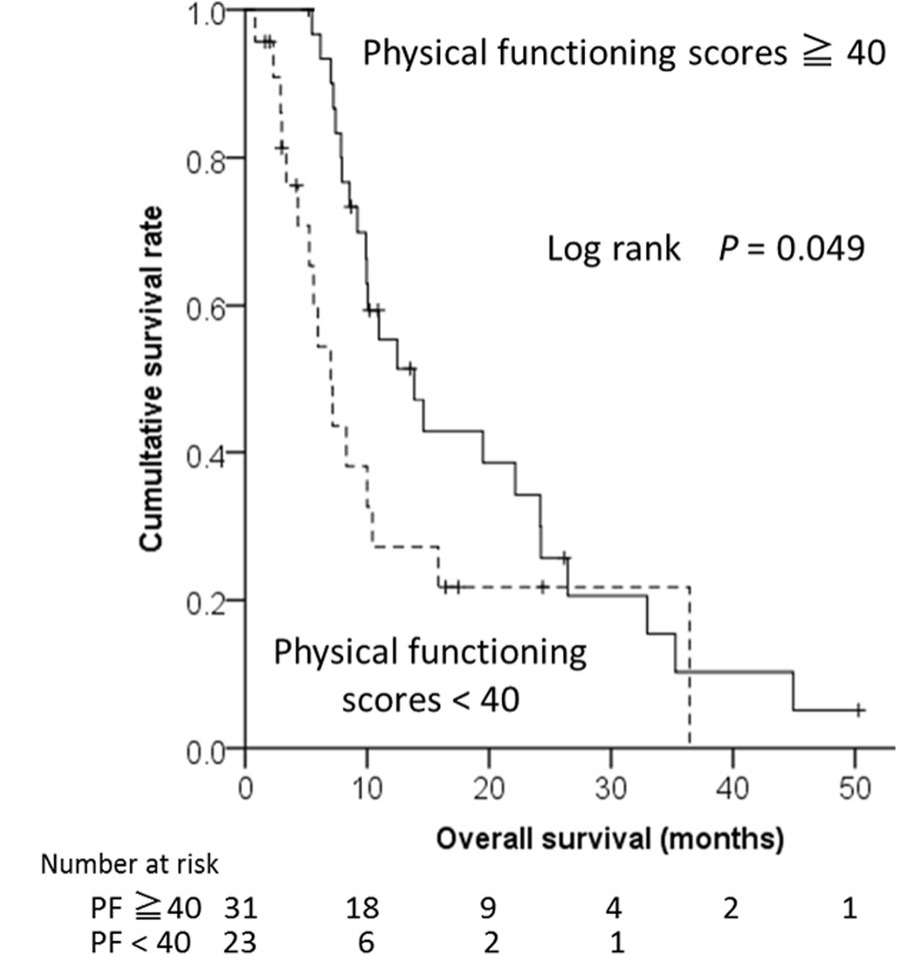
Kaplan-Meier graphs displaying time-to-death stratified by factors associated with overall survival: physical functioning (PF) scores

The median overall survival was 9.6 (IQR: 0.8–16.3) months. Previous curative therapy and a physical functioning domain score ≥40 at baseline were associated with longer overall survival by multivariate analysis (Table 2 and Fig. 2).

**Table 2:** Baseline demographic and clinical variables and baseline domain scores associated with overall survival

Variable	Univariate^a^		Multivariate^a^	
	HR (95 % CI)	*P*	HR (95 % CI)	*P*
Baseline characteristics				
Age <70 y (vs. ≥70 y)	0.603 (0.349–1.258)	0.208		
Sex, male (vs. female)	0.544 (0.254–1.167)	0.118		
HCV infection (vs. other etiology)	1.138 (0.610–0.122)	0.684		
Child Pugh = 5 (vs. ≥6)	0.419 (0.227–0.812)	0.009		
TMN stage III (vs. IV)	0.695 (0.371–1.304)	0.257		
Vascular invasion - (vs. +)	0.475 (0.204–0.945)	0.034		
Tumor size <50 mm (vs. ≥50 mm)	0.831 (0.441–1.567)	0.567		
Previous curative therapy: Yes (vs. No)	0.255 (0.129–0.504)	<0.001	0.235 (0.116–0.477)	<0.001
AFP <100 (vs. ≥100)	0.893 (0.473–1.686)	0.726		
DCP <1000 (vs. ≥1000)	0.548 (0.280–1.070)	0.078		
Initial dose of sorafenib 800 mg (vs. <800 mg)	1.137 (0.611–2.115)	0.686		
Treatment duration ≥5 months (vs. <5)	0.608 (0.325–1.138)	0.120		
HRQOL domain scores ≥40^b^:				
Physical functioning	0.529 (0.278–1.008)	0.053	0.479 (0.245–0.935)	0.031
Role physical	0.595 (0.314–1.127)	0.111		
Bodily pain	1.686 (0.772–3.676)	0.190		
General health	1.331 (0.644–2.755)	0.440		
Vitality	0.842 (0.383–1.848)	0.669		
Social functioning	0.833 (0.411–1.684)	0.610		
Role emotional	0.957 (0.473–1.938)	0.904		
Mental health	2.053 (0.893–4.717)	0.090		

*HR* hazard ratio, *CI* confidence interval, *HCV* hepatitis C virus, *TNM* tumor-node-metastasis, *AFP* alpha fetoprotein, *DCP* des-gamma-carboxy prothrombin, *HRQOL* health related quality of life

^a^Cox proportional hazards regression analysis

^b^All domain scores are relative to scores <40

All variables with *P* values <0.06 in the univariate analysis were included in the multivariate analysis

**Fig. 3:**
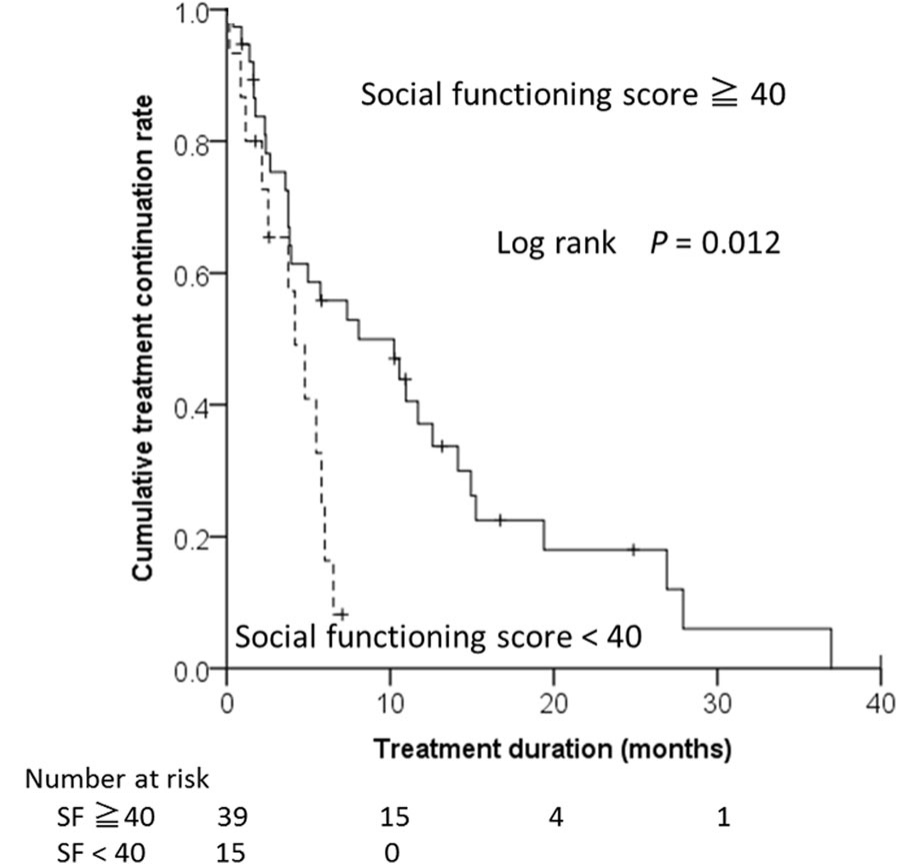
Kaplan-Meier graphs displaying treatment duration stratified by social functioning (SF) scores

Age, liver function (Child-Pugh score), and HCC stage were not significantly associated with overall survival, neither were serum alpha-fetoprotein or DCP levels. The median duration of sorafenib treatment was 4.9 (IQR: 0.2–10.8) months. Social functioning domain scores ≥40 at baseline, absence of vascular invasion, and lower DCP values were significant predictors for longer treatment duration (Table 3 and Fig. 3). The association of social functioning domain scores with treatment duration was confirmed by the competing risk analysis (Additional file 1: Figure S4).

**Table 3:** Baseline demographic and clinical variables and domain scores associated with treatment duration

Variables	Univariate^a^		Multivariate^a^	
	HR (95 % CI)	*P* value	HR (95 % CI)	*P* value
Baseline characteristics				
Age <70 y (vs. ≥70 y)	0.749 (0.343–1.429)	0.380		
Sex, male (vs. female)	0.634 (0.307–1.308)	0.217		
HCV infection (vs. other etiology)	1.796 (0.964–3.348)	0.065		
Child Pugh = 5 (vs. ≥6)	0.511 (0.271–0.962)	0.038		
TMN stage III (vs. IV)	0.672 (0.357–1.264)	0.218		
Vascular invasion – (vs. +)	0.355 (0.178–0.710)	0.003	0.382 (0.186–0.786)	0.009
Tumor size <50 mm (vs. ≥50 mm)	0.777 (0.412–1.466)	0.432		
Previous curative therapy: Yes (vs. No)	0.433 (0.226–0.829)	0.012		
AFP <100 (vs. ≥100)	1.048 (0.561–1.961)	0.954		
DCP <1000 (vs. ≥1000)	0.473 (0.509–0.912)	0.025	0.509 (0.261–0.993)	0.048
Initial dose of sorafenib 800 mg (vs. <800 mg)	0.744 (0.388–1.426)	0.373		
HRQOL domain scores ≥40:^b^				
Physical functioning	0.956 (0.509–1.799)	0.889		
Role physical	0.771 (0.416–1.429)	0.408		
Bodily pain	1.582 (0.728–3.436)	0.247		
General health	1.034 (0.516–2.070)	0.926		
Vitality	1.534 (0.631–3.731)	0.346		
Social functioning	0.395 (0.185–0.840)	0.016	0.452 (0.206–0.995)	0.049
Role emotional	0.898 (0.455–1.770)	0.755		
Mental health	0.833 (0.379–1.828)	0.649		

*HR* hazard ratio, *CI* confidence interval, *HCV* hepatitis C virus, *TNM* tumor-node-metastasis, *AFP* alpha fetoprotein, *DCP* des-gamma-carboxy prothrombin; HRQOL, health related quality of life

^a^Cox proportional hazards regression analysis

^b^All domain scores are relative to scores <40

All variables with *P* values <0.05 in the univariate analysis were included in the multivariate analysis

## Discussion

HRQOL was not significantly impaired in the patients who could receive sorafenib treatment over the course of 1 year (Additional file 1: Figure S2 and Table S2). A previous study showed decreased HRQOL domain scores in the 2 months after initiation of sorafenib in patients with advanced HCC because of adverse events [13]. In contrast, sorafenib treatment did not significantly affect HRQOL in patients with metastatic renal cell carcinoma [9, 21]. We found no significant differences in HRQOL domain scores between patients who experienced grade 3 adverse effects and those who did not (Additional file 1: Figure S3). The severe adverse effects associated with sorafenib use, reported to occur in 50 % of patients [22], potentially influence HRQOL. Interestingly, skin toxicity [23], hypertension [24], and diarrhea [25] are known to be associated with better prognosis in sorafenib therapy. Therefore, the substantial anti-tumor effects of sorafenib might have countered severe adverse effects both physically and mentally.

Within HRQOL, physical functioning, role limitations because of physical problems (RP), and vitality domain scores gradually decreased towards death. These declines would have reflected the progression of HCC and the deterioration of underlying liver diseases. This is the first prospective long term follow-up study on HRQOL scores in patients with advanced HCC receiving sorafenib therapy. Interestingly, scores in the psychosocial HRQOL domains—social functioning, role limitations because of emotional problems (RE), and mental health—were maintained >40 points until death. The bodily pain score also remained >40, suggesting that the psychosocial domains and bodily pain can be managed well by periodical medical and nursing interventions. Higher physical functioning domain scores at baseline were significantly associated with longer overall survival. Our study also demonstrated that physical functioning decreased significantly towards death. Taken together with the results of Cox regression analysis, physical functioning domain scores could be good predictors of prognosis. These results are in accordance with previous studies, which revealed better physical functioning and role limitations because of physical problems as predictors for longer survival in patients with non-resectable HCC [26] and in those requiring palliative care for HCC [27]. Previous studies suggest that daily physical activity contributes to the decrease in mortality due to liver cancer [28, 29]. A prospective study is necessary to verify whether adequate levels of physical activity would improve the prognosis of HCC patients. Our study, for the first time, showed that HRQOL domain scores were useful to predict prognosis in patients with advanced HCC receiving sorafenib. Thus, when we observe a decline in physical functioning domain scores, we should pay more careful attention to the patient’s condition. These observations are supported by studies of frailty. Frailty is significantly associated with depression [30] and mortality [31] in patients with end-stage liver disease. The impact of frailty on mortality is evident in elderly patients [32]. Given that the Japanese patients with HCC are mostly geriatric (median age in this study: 71 years old), the concept of frailty appears important to predict prognosis [33].

Higher social functioning domain scores at baseline contributed to longer treatment duration. The social functioning domain score might reflect social support associated with adherence to sorafenib. Sufficient social support from the beginning of sorafenib therapy would be helpful to continue medication, which is potentially accompanied by severe adverse effects. We also found that the presence of vascular invasion and a DCP value >1000 mAU/mL were significant predictors of shorter treatment duration. Tumors with vascular invasion [34] or an associated high level of DCP [35] are likely to have an aggressive phenotype.

Many studies associate preserved liver function with better survival [36], however, Child-Pugh score as an index of liver function was not chosen as a significant predictor for overall survival in this study. The reason for this discrepancy can be explained by the background of our cohort; liver function was well preserved in most patients (Child-Pugh score 5: 59 %, score 6: 32 %, and score 7: 9 %).

Different etiologies can influence the interpretation of the results of clinical studies. The major causes of HCC in this cohort were HCV (44 %), HBV (20 %), and alcohol (13 %). A similar trend is seen in Europe and North America [37], where the leading causes of HCC are HCV (50–70 %), HBV (20–30 %), and alcohol (20 %). Hence, our results could be applicable to such areas.

The presence of previous curative therapy was significantly associated with better overall survival. The difference in the biological nature of tumors may explain these results. Tumors that recur after curative treatment may be less aggressive than other tumors.

There are some limitations to this study. Although the study was prospective, it was conducted in a single institution with a relatively small number of patients. The initial dose of sorafenib used in our study was 800 mg, but lower doses were used for some elderly patients. Thus, initial doses were relatively low compared with those used in a previous study [38]. However, the initial dose did not affect prognosis in the present study. Nevertheless, we cannot deny a potential influence of initial sorafenib dose on overall survival and treatment duration.

## Conclusions

In conclusion, HRQOL was not significantly impaired in patients who could receive sorafenib treatment over the course of 1 year. Physical functioning scores ≥40 and social functioning scores ≥40 at baseline were significantly associated with longer overall survival and longer treatment duration, respectively. Thus, HRQOL could be a valuable marker to predict the clinical course of patients with advanced HCC receiving sorafenib.

## Additional file

Additional file 1: Table S1. Changes in HRQOL domain scores in the 12 months prior to death. **Table S2**. Changes in HRQOL domain scores in patients who survived >1 year (*n* = 13). **Figure S1**. Graphical display of the distribution of sorafenib-related adverse events (*n* = 54). **Figure S2**. Graphical representation of HRQOL domain score changes of patients receiving sorafenib over the course of one year (*n* = 13). **Figure S3**. Graphical representation of HRQOL domain score changes with/without grade 3 adverse events. **Figure S4**. Cumulative discontinuation incidence curves stratified by factors associated with treatment duration: social functioning (SF) scores. (DOCX 553 kb)

## Abbreviations

AFP: Alpha fetoprotein
BP: Bodily pain
CT: Computed tomography
DCP: Des-gamma-carboxy prothrombin
GH: General health
HCC: Hepatocellular carcinoma
HCV: Hepatitis C virus
HRQOL: Health-related quality of life
IQR: Interquartile range
MH: Mental health
MRI: Magnetic resonance imaging
PF: Physical functioning
RE: Role emotional
RP: Role physical
SF: Social functioning
TNM: Tumor-node-metastasis
VT: Vitality
