# Supplementary material for: Longitudinal alterations in health-related quality of life and its impact on the clinical course of patients with advanced hepatocellular carcinoma receiving sorafenib treatment
Source: BMC Cancer. 2016 Nov 11;16:878. doi: 10.1186/s12885-016-2908-7 (PMC5106792; doi:10.1186/s12885-016-2908-7)
Supplement: Additional file 1: Table S1. — Changes in HRQOL domain scores in the 12 months prior to death. Table S2. Changes in HRQOL domain scores in patients who survived >1 year (n = 13). Figure S1. Graphical display of the distribution of sorafenib-related adverse events (n = 54). Figure S2. Graphical representation of HRQOL domain score changes of patients receiving sorafenib over the course of one year (n = 13). Figure S3. Graphical representation of HRQOL domain score changes with/without grade 3 adverse events. Figure S4. Cumulative discontinuation incidence curves stratified by factors associated with treatment duration: social functioning (SF) scores. (DOCX 553 kb) [file 12885_2016_2908_MOESM1_ESM.docx]

**Additional file 1**

**Table S1.** Changes in HRQOL domain scores in the 12 months prior to death

| Domain | PF | RP | BP | GH | VT | SF | RE | MH |
| --- | --- | --- | --- | --- | --- | --- | --- | --- |
| KW test | 0.000 | 0.008 | 0.981 | 0.012 | 0.000 | 0.798 | 0.442 | 0.048 |
| Mann-Whitney U test | | | | | | | | |
| 0 m vs. 3 m | 0.001 | 0.062 | 0.829 | 0.100 | 0.006 | 0.670 | 0.381 | 0.044 |
| 0 m vs. 6 m | 0.001 | 0.009 | 0.717 | 0.014 | 0.012 | 0.305 | 0.114 | 0.120 |
| 0 m vs. 9 m | 0.000 | 0.032 | 0.714 | 0.002 | 0.000 | 0.488 | 0.170 | 0.007 |
| 0 m vs. 12 m | 0.007 | 0.003 | 0.967 | 0.088 | 0.002 | 0.376 | 0.248 | 0.117 |
| 3 m vs. 6 m | 0.638 | 0.276 | 0.636 | 0.437 | 0.871 | 0.533 | 0.519 | 0.921 |
| 3 m vs. 9 m | 0.192 | 0.539 | 0.596 | 0.086 | 0.095 | 0.677 | 0.565 | 0.291 |
| 3 m vs. 12 m | 0.312 | 0.090 | 0.699 | 0.508 | 0.038 | 0.556 | 0.583 | 0.486 |
| 6 m vs. 9 m | 0.683 | 0.647 | 0.935 | 0.235 | 0.076 | 0.920 | 0.947 | 0.241 |
| 6 m vs. 12 m | 0.493 | 0.463 | 0.962 | 0.888 | 0.030 | 0.737 | 1.000 | 0.312 |
| 9 m vs. 12 m | 0.606 | 0.335 | 0.973 | 0.657 | 0.212 | 0.781 | 0.832 | 0.891 |

HRQOL, health-related quality of life; PF, physical functioning; RP, role physical; BP, bodily pain; GH, general health; VT, vitality; SF, social functioning; RE, role emotional; MH, mental health; KW test, Kruskal Wallis test.

0 m, imminent death (n=40): 3 m, 3 months before death (n=31); 6 m, 6 months before death (n=22); 9 m, 3 months before death (n=14); 12 m, 12 months before death (n=8).

**Table S2.** Changes in HRQOL domain scores in patients who survived >1 year (n=13)

| **Domain** | **PF** | **RP** | **BP** | **GH** | **VT** | **SF** | **RE** | **MH** |
| --- | --- | --- | --- | --- | --- | --- | --- | --- |
| Friedman | 0.075 | 0.549 | 0.338 | 0.579 | 0.262 | 0.677 | 0.285 | 0.486 |
| Wilcoxon two-sample test | | | | | | | | |
| BL vs. 3 m | 0.164 | 0.575 | 0.213 | 0.366 | 0.059 | 0.397 | 0.283 | 0.126 |
| BL vs. 6 m | 0.189 | 0.834 | 0.286 | 0.889 | 0.158 | 0.674 | 0.638 | 0.207 |
| BL vs. 9 m | 0.075 | 0.505 | 0.583 | 0.814 | 0.310 | 0.799 | 0.894 | 1.000 |
| BL vs. 12 m | 0.624 | 0.505 | 0.272 | 0.248 | 0.824 | 0.476 | 0.767 | 0.576 |
| 3 m vs. 6 m | 0.767 | 0.953 | 0.859 | 0.475 | 0.583 | 0.759 | 0.779 | 0.799 |
| 3 m vs. 9 m | 1.000 | 0.397 | 0.508 | 0.556 | 0.878 | 0.362 | 0.753 | 0.327 |
| 3 m vs. 12 m | 0.130 | 0.929 | 0.065 | 0.814 | 0.133 | 0.507 | 0.063 | 0.278 |
| 6 m vs. 9 m | 1.000 | 0.140 | 0.415 | 1.000 | 0.656 | 0.866 | 0.933 | 0.248 |
| 6 m vs. 12 m | 0.071 | 0.211 | 0.031 | 0.114 | 0.050 | 0.050 | 0.314 | 0.114 |
| 9 m vs. 12 m | 0.109 | 0.284 | 0.508 | 0.100 | 0.049 | 0.310 | 0.181 | 0.255 |

HRQOL, health-related quality of life; Friedman, Friedman test and Bonferroni correction; BL baseline; 3, 6, and 9 m; 3, 6, and 9 months post-initiation of sorafenib; PF, physical functioning; RP, role physical; BP, bodily pain; GH, general health; VT, vitality; SF, social functioning; RE, role emotional; MH, mental health.

**Figure S1**. **Graphical display of the distribution of sorafenib-related adverse events (n = 54)**

All values represent the percentages of the occurrence of adverse events.

All value shows the percentages of the occurrence of adverse events.

**Figure S2. Graphical representation of HRQOL domain score changes of patients receiving sorafenib over the course of one year (n=13)**

HRQOL, health-related quality of life; BL, baseline; 3M, 3 months; 6M, 6 months; 9M, 9 months; 12M, 12 months; PF, physical functioning; RP, Role physical; BP, bodily pain; GH, general health; VT, vitality; SF, social functioning; RE, Role emotional; MH, mental health


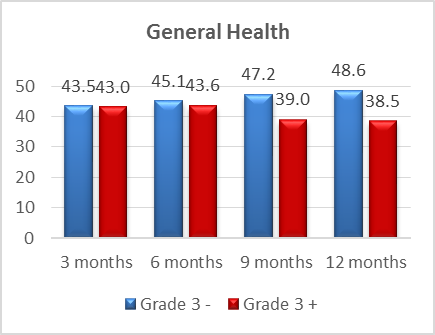

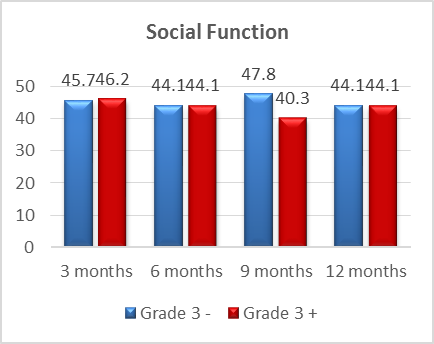

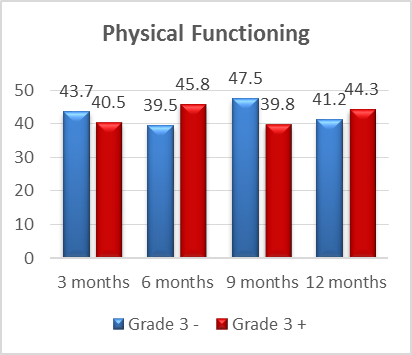

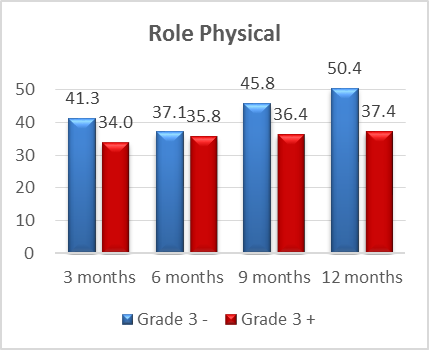

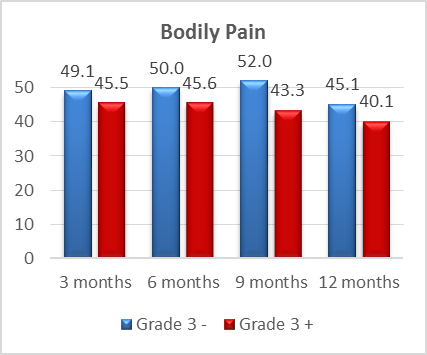

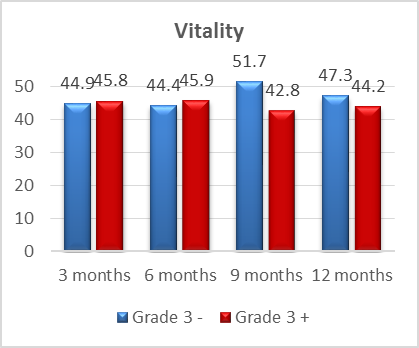

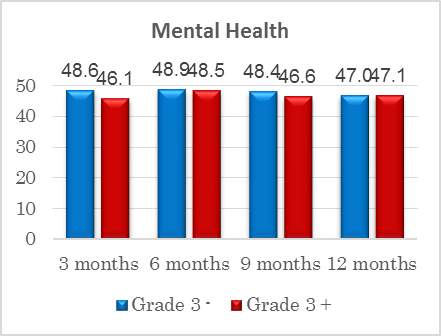

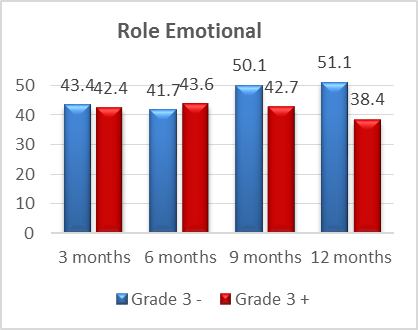


**Figure S3.** **Graphical representation of HRQOL domain score changes with/without grade 3 adverse events**

Abbreviations: 3, 6, 9, and 12 months shows the time from initiation of sorafenib.

Grade 3 -; no grade 3 adverse events during sorafenib therapy.

Grade 3 +; experienced grade 3 adverse events during sorafenib therapy.

*3 months (n=42); 6 months (n=31); 9 months (n=22); 12 months (n=13).

**All domain scores showed no significant differences by Kruskal-Wallis test and Mann-Whitney U test.


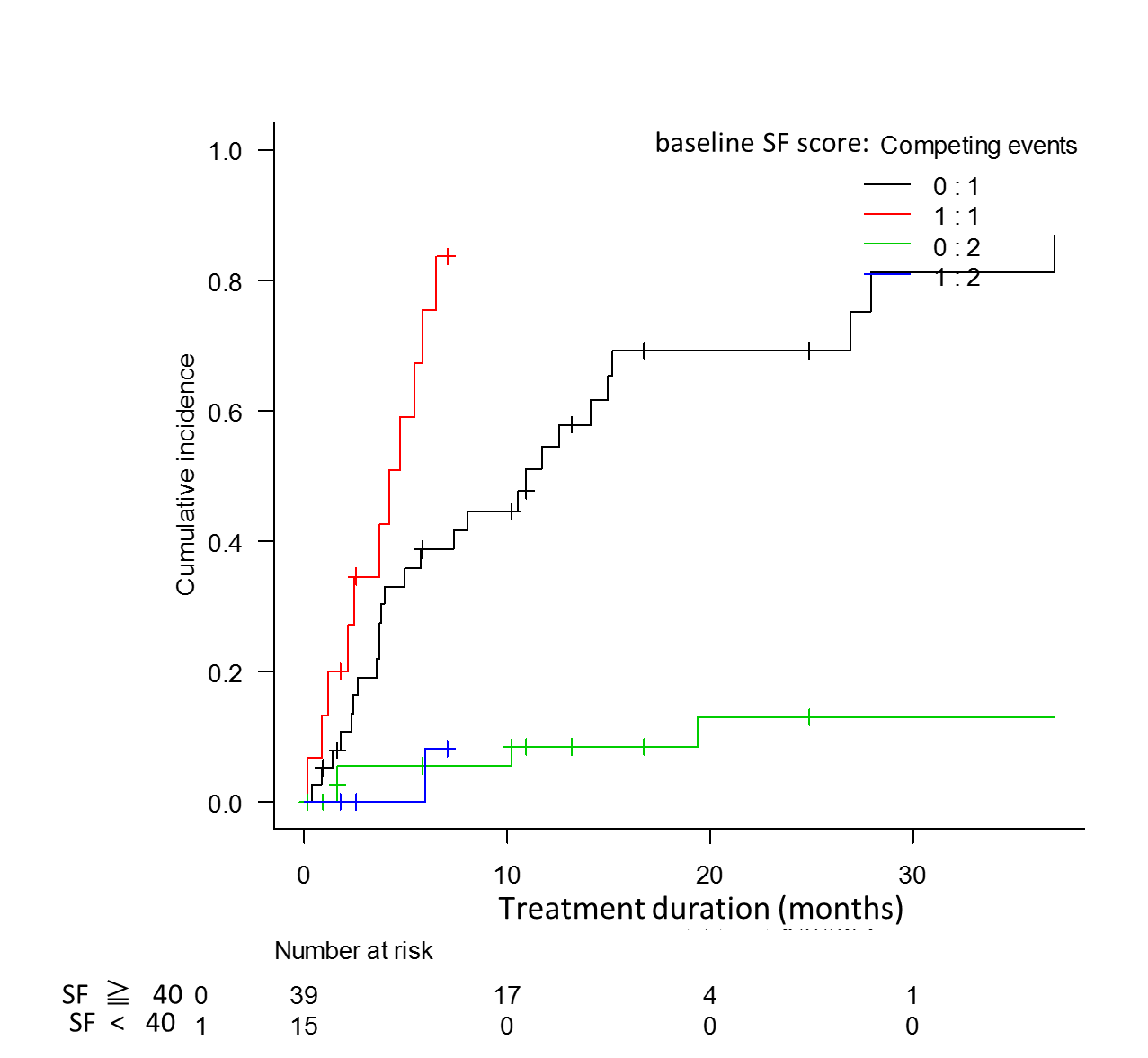


**Figure S4. Cumulative discontinuation incidence curves stratified by factors associated with treatment duration: social functioning (SF) scores**

* These curves were developed and compared using competing risk analysis, Gray’s method.

Cumulative incidence of sorafenib discontinuation when death is a competing risk: Black line, baseline SF score ≥40; Red line, baseline SF score <40; p=0.0112.

Cumulative incidence of death when discontinuation of sorafenib is a competing risk: Green line, baseline SF ≥40; Blue line, baseline SF <40; p=0.864.

Abbreviations: SF; social functioning
